# Correlation between the Intensity of *Helicobacter pylori* Colonization and Severity of Gastritis

**DOI:** 10.1155/2017/8320496

**Published:** 2017-11-28

**Authors:** Hamid Reza Ghasemi Basir, Mehdi Ghobakhlou, Parvin Akbari, Arash Dehghan, Mohamad Ali Seif Rabiei

**Affiliations:** ^1^Department of Pathology, School of Medicine, Hamadan University of Medical Sciences, Hamadan, Iran; ^2^Clinical Research Development Unit of Shahid Beheshti Hospital, Hamadan University of Medical Sciences, Hamadan, Iran; ^3^School of Medicine, Hamadan University of Medical Sciences, Hamadan, Iran; ^4^Department of Community Medicine, School of Medicine, Hamadan University of Medical Sciences, Hamadan, Iran

## Abstract

**Background:**

The most common cause of chronic gastritis is infection with *Helicobacter pylori*. Identifying the relationship between intensities of colonization and activity of gastritis helps the clinician in more effective treatment and posttreatment follow-ups.

**Methods:**

In this cross-sectional study, endoscopic gastric biopsy samples of 544 patients who complained symptoms of dyspepsia for more than three months referring to the laboratory were studied. To determine the colonization rate of *H. pylori* and other pathological findings, Giemsa and H&E stains were, respectively, used.

**Results:**

Among 544 subjects, 47 (8.64%) patients had no gastritis, 203 (37.32%) had mild gastritis, 278 (10.51%) suffered moderate gastritis, and 16 (2.94%) had severe gastritis. In this study, patients with mild *H. pylori* colonization rates had the highest level of mild activity (33.52%); in contrast, those with severe *H. pylori* colonization had the highest level of severe activity (43.75%). 93.96% of people with severe *H. pylori* colonization suffered from moderate and severe chronic gastritis. There is a significant statistical relationship between the intensity of *H. pylori* colonization and histopathological findings including intestinal metaplasia, atrophy, and lymphoid follicle formation.

**Conclusions:**

According to the present study, with increasing intensity of *H. pylori* colonization, chronicity and activity of gastritis and its complications increase.

## 1. Introduction

Today, the most common cause of chronic gastritis is infection with *Helicobacter pylori* (*H. pylori*). In addition to active chronic gastritis, this organism has a strong relationship with gastric adenocarcinoma and MALToma [1]. Causes of gastritis are widespread and heterogeneous. Gastritis can be classified according to the duration of the disease (acute or chronic), histological aspects, anatomical distributions, and possible pathogenic mechanisms. Histologically, chronic gastritis is characterized by infiltration of inflammatory cells with the preference of lymphocytes and plasma cells, along with a number of neutraophils, in the lamina propria. Chronic gastritis has three stages. In the first stage, inflammatory changes are limited to the laminar propria of the mucosal surface together with edema and inflammatory infiltration which separate the normal-appearing gastric glands. In the second stage, inflammatory infiltration extends to the deeper areas of the mucosa and is associated with deformation and destruction of the glands. In the third stage, the gland structures are demonstrating atrophic changes and inflammatory infiltrations decrease. Atrophic gastritis along with metaplasia is observed in chronic gastritis due to *Helicobacter pylori*. This may ultimately lead to gastric adenocarcinoma [2].

*Helicobacter pylori* is a microaerophilic gram-negative bacillus that is commonly found in the deep parts of the mucous gel covering the gastric mucosa or between the mucous layer and the gastric epithelium. The prevalence of *H. pylori* is higher in older ages. Two prone factors to increase the colonization of this organism include poor socioeconomic status and low education. Nature of the inflammatory response created by *H. pylori* is defined based on bacterial virulence factors, intensity of bacterial colonization, and the host response [3].

Chronic infection with *H. pylori* stimulates the host's immune response, causes active chronic inflammation and mucosal injury resulting in multifocal atrophic gastritis and intestinal metaplasia, glandular dysplasia, and adenocarcinoma [4]. After treatment, the shape of the bacteria may also become round or *Vibrio* and *H. pylori* colonization decreases or reaches zero. After eradication of *H. pylori*, histology of the lesion is improved [5].

Considering the importance and prevalence of gastritis, the relationship between intensities of gastritis and bacterial colonization is investigated; and the necessity of specific staining of *Helicobacter pylori* is determined according to the results. On the other hand, identifying the relationship between intensities of colonization and gastritis helps the clinician in more effective treatment and posttreatment follow-ups (response rate to antibiotic therapy).

## 2. Methodology

In this cross-sectional study, endoscopic gastric biopsy samples of 544 patients referring to the Razi laboratory of Hamadam city in Iran were studied between 2014-2015 regardless of their occupation, education, economic and social status, and food habits. The mentioned patients complained symptoms of dyspepsia for more than three months and had not used antibiotics during the past month; they had no history of surgery on the gastrointestinal tract and did not have any underlying disease other than dyspepsia. To determine the colonization rate of *H. pylori* and other pathological findings, Giemsa and H&E stains were, respectively, used. In this study, we used the Sydney system grading of chronic gastritis for grading of chronicity, activity, and *H. pylori* density [6]. Scattered organisms covering less than one third of the surface are regarded as mild colonization; large clusters or a continuous layer over two thirds of the surface is graded as severe; intermediate numbers are mentioned as moderate colonization. The normal number of gastric mucosal mononuclear cells in the lamina propria is defined as a maximum of 2 to 5 lymphocytes, plasma cells, and macrophages per high-power field (×40 objective). Mild chronic inflammation is defined as a mild increase of inflammatory infiltration, predominantly plasma cells, within the lamina propria in a patchy, loose distribution without destruction or involvement of epithelium using ×10 objective lens to identify mononuclear clusters. Dense lymphoplasma cell infiltration of the lamina propria with or without lymphoid follicles, easily identifiable on ×4 objective lens, with infiltration and destruction of epithelium is regarded as severe chronic inflammation. Intermediate status is mentioned as moderate degree. Activity of gastritis is defined as neutrophilic infiltration of the lamina propria, pits, or surface epithelium. Less than one third of pits and surface infiltration are regarded as mild; one third to two thirds are graded as moderate; more than two thirds are regarded as severe.

Atrophic changes are defined as loss of specialized glands from either the antrum or corpus. Metaplastic epithelium is recognized morphologically by the presence of goblet cells, absorptive cells, and cells resembling colonocytes.

Patient information was entered into the checklist and analyzed with SPSS16. The significance level was determined as 0.05; amounts less than five hundredths were considered statistically significant. The statistical test that is used was chi-square.

## 3. Findings

The average age of the participants in the study was 43.62 years with a standard deviation of 16.74 with a minimum and maximum age of 1 and 87 years (with an age range of 86 years), respectively. The average age for men and women was 45.87 and 41.50 years, respectively.

Of the 544 patients with *H. pylori* infection, 51.84% were female and there was no statistically significant relationship between gender and *H. pylori* infection (*P* value = 0.67). Individuals with mild, moderate, and severe *H. pylori* infection were divided by gender in which 52%, 54%, and 43% of them are female, respectively. Despite the higher percentage of women in the mild and moderate infected groups, there was no statistically significant relationship between gender and intensity of *H. pylori* infection (*P* value = 0.56).

Among 544 subjects, 47 (8.64%) patients had no gastritis, 203 (37.32%) had mild gastritis, 278 (51.10%) suffered from moderate gastritis, and 16 (2.94%) had severe gastritis.

Most of the participants were in the mild activity group and the moderate activity group included the least number of patients (Table 1).

15.07% the participants had superficial erosion. 4.23% had ulcer, 6.07% had atrophy, and 12.50% showed metaplasia. Also, 46.11% of the biopsies revealed lymphoid follicles.

64.15% of the participants suffered from mild *H. pylori* infection, while 29.96% and 5.88% of the patients had moderate and severe *H. pylori* colonization, respectively.

In this study, patients with mild *H. pylori* colonization rates had the highest level of mild activity (33.52%); in contrast, those with severe *H. pylori* colonization had the highest level of severe activity (43.75%). There was a statistically significant relationship between the severity of *H. pylori* infection and activity (*P* value < 0.001). Hence, the level of activity increases by increased intensity of colonization (Table 2).

In this study, the severity of chronic gastritis was mostly reported as mild (51.58%) in people with mild *H. pylori* colonization. In contrast, 93.76% of people with severe *H. pylori* colonization suffered from moderate and severe chronic gastritis. There was a significant statistical relationship between the incidence of *H. pylori* infection and the severity of chronic gastritis. As shown in Table 3, the severity of chronic gastritis increases with the increasing intensity of *H. pylori* colonization.

As Table 4 shows, there is a significant statistical relationship between the intensity of *H. pylori* colonization and histopathological findings including intestinal metaplasia, atrophy, and lymphoid follicle formation. However, no significant relationship was found between the intensity of colonization and ulcer formation. That is, by increasing the degree of colonization, likelihood of the formation of metaplasia, atrophy, and lymphoid follicle formation is increased.

## 4. Discussion

Regarding its numerous complications affecting the upper gastrointestinal tract and its high prevalence in developing countries including our country, correct diagnosis and proper treatment of *H. pylori* infection are a necessity. The aim of this study was to investigate the relationship between the intensity of *H. pylori* colonization in gastric mucosa and the severity of gastric mucosal pathological findings in biopsy specimens.

In this study, more than 90% of people infected with *H. pylori* experienced varying degrees of gastritis, with moderate gastritis (51.10%) having the highest incidence. In the study conducted by Hashemi et al. in southern Iran (2006), 70% of people infected with *H. pylori* suffered from chronic gastritis and in another study in Saudi Arabia, this rate was 60 percent [7, 8]. Compared to these studies, the incidence of chronic gastritis secondary to *H. pylori* infection is higher in our study due to the evaluation of symptomatic patients.

The findings of this study showed that there is a dose response relationship between *H. pylori* colonization and activity such that in mild colonization, only 20.63% of the samples had severe activity while in intense colonization of *H. pylori*, 43.75% of the samples had severe activity. In a study by Alagoz et al. in Turkey (2002), it was shown that there is a positive correlation between the level of colonization and activity (Pearson correlation coefficient = 0.72) which was confirmed by the present study as well [9].

Our study also showed that there is a direct relationship between the severity of *H. pylori* infection and the degree of chronic gastritis. Hence, if the level of colonization is mild, the chance of severe chronic gastritis among the subjects was only 0.57% while this chance was about 28 percent among those with severe colonization.

In a study conducted by Ardakani and Mohammadizadeh in Isfahan (2006) on 272 samples of gastric biopsy, there was no significant relationship between the density and volume of *H. pylori* and the severity of chronic gastritis activity [3].

In a study by Yakoob and Hussainy in Pakistan (2010), there was a statistically significant correlation between the intensity of *H. pylori* colonization and chronic gastritis activity [10].

This relationship was not found in the study conducted by Park et al. in Korea, which could be due to the genetic differences, nutritional habits, and environmental factors between the two study populations [11].

The study of Choudhary et al. in Nepal (2001) entitled “Correlation of H. Pylori density with grading of chronic gastritis” conducted on 251 patients showed a lack of correlation [12]. However, as already mentioned, a coherent correlation was observed in our study.

In this study, there was no statistically significant difference between the levels of *H. pylori* infection by gender. Albeit, the prevalence of different degrees of *H. pylori* infection was higher in women except for severe infection which may be due to frequent visits by women to the clinic for treatment. In this study, 48.16% of the subjects were male, while the female population was 51.84%. In most previous studies, there was no significant relationship between *H. pylori* infection and gender. For example, the results of a study conducted by Everhart et al. in 2000 in the United States showed that there is no statistically significant difference between two genders [13]. Findings of our study were consistent with the study by Klein et al. (1994) regarding gender differences [14].

In this study, the percentage of intestinal metaplasia observed in 68 subjects was 12.5%, which is higher than the percentage reported by Fakher.Yasseri H. (11.3%) [15]. In the study of Zhang et al. in Japan (2005), 37% of the subjects with chronic gastritis had metaplasia [16].

The results of a study conducted in Iraq in 2011 indicated a higher prevalence of intestinal metaplasia compared to our study. In this study, the prevalence of metaplasia was 15%, while this rate was 12.5% in our study [17]. Perhaps the high percentage of metaplasia in other studies is due to genetic differences, nutritional habits, and differences in the duration of gastritis.

In this study, there was no significant relationship between *H. pylori* colonization and ulcer development.

In our study, only 23 cases (23.24%) suffered from ulcer among pathology specimens. There was no statistically significant relationship between ulcer and *H. pylori* colonization severity. In mild, moderate, and severe colonization, 2.87%, 6.13%, and 9.38%, respectively, had ulcer.

In a study by Zhang et al. in Japan in 2005, it was concluded that there is a strong correlation between the amount of *H. pylori* colonization and ulcer [16]. This difference might be due to the small number of people with ulcer in our study.

The results of our study showed that 33 patients (6.07%) had atrophic disease. Among pathologic specimens, 231 cases (46.11%) of lymphoid follicles were observed. In both conditions, there was a statistically significant difference between the intensity of colonization in those with and without such complications. The study results of Kuipers et al. (1995) in the Netherlands confirmed the findings of our study, suggesting that *H. pylori* infection is an important risk factor for atrophy and the formation of lymphoid follicles [18].

One of the strengths of our study is its large sample volume compared to other studies. If this study was simultaneously conducted at multicenters, it could have a higher ability to conclude on hypotheses.

## 5. Conclusion

According to the present study, with increasing intensity of *H. pylori* colonization, chronicity and activity of gastritis and its complications and the consequent financial burdens imposed on the health system may increase. Correct treatment of *H. pylori* infection can be useful in preventing chronic gastritis and its complications. Therefore, it seems that reduction of *H. pylori* colonization should be considered as a health goal.

## Figures and Tables

**Table 1:** Frequency distribution of the activity level of inflammation among the subjects participating in the study.

Activity level of inflammation	Frequency	Percentage
Without activity	166	30.51
Mild	201	36.95
Moderate	66	12.13
Severe	111	20.42
Total	544	100

**Table 2:** Relationship between the level of activity and severity of *H. pylori* infection in patients with chronic gastritis.

		Activity intensity				
		Lack of activity	Mild	Moderate	Severe	Total
The severity of *Helicobacter pylori* colonization	Mild	142 (40.69%)	117 (33.52%)	18 (5.16%)	72 (20.63%)	349 (100%)
	Moderate	24 (14.72%)	73 (44.79%)	41 (25.15%)	25 (15.34%)	163 (100%)
	Severe	0 (0.00%)	11 (34.38%)	7 (21.88%)	14 (43.75%)	32 (100%)
	Total	166 (30.51%)	201 (36.95%)	66 (12.13%)	111 (20.40%)	544 (100%)

*P* value (chi-square) < 0.001.

**Table 3:** Relationship between severity of chronic gastritis and intensity of *H. pylori* infection.

		Chronic gastritis severity				
		Without gastritis	Mild	Moderate	Severe	Total
The severity of *Helicobacter pylori* colonization	Mild	46 (13.18%)	180 (51.58%)	121 (34.67%)	2 (0.57%)	349 (100%)
	Moderate	1 (0.61%)	21 (12.88%)	136 (83.44%)	5 (3.07%)	163 (100%)
	Severe	0 (0.00%)	2 (6.25%)	21 (65.63%)	9 (28.13%)	32 (100%)
	Total	47 (8.64%)	203 (37.32%)	278 (51.10%)	16 (2.94%)	544 (100%)

*P* value (chi-square) < 0.001.

**Table 4:** Relationship between histopathological findings and severity of *H. pylori* infection in patients with chronic gastritis.

The severity of *Helicobacter pylori* colonization			Mild	Moderate	Severe	*P* (chi-square)
Histopathological findings	Metaplasia	Yes	30 (8.60%)	32 (19.63%)	6 (18.75%)	0.001
		No	319 (91.40%)	131 (80.37%)	26 (81.25%)	
	Atrophy	Yes	13 (3.72%)	15 (9.20%)	5 (15.63%)	0.004
		No	336 (96.28%)	148 (90.80%)	27 (84.38%)	
	Ulcer	Yes	10 (2.87%)	10 (6.13%)	3 (9.38%)	0.07^∗^
		No	339 (97.13%)	153 (93.87%)	29 (90.63%)	
	Lymphoid follicle	Yes	106 (34.53%)	100 (61.73%)	25 (78.13%)	0.001
		No	201 (65.47%)	62 (38.27%)	7 (21.88%)	

^∗^Not significant.
